# Clove Essential Oil (*Syzygium aromaticum* L. Myrtaceae): Extraction, Chemical Composition, Food Applications, and Essential Bioactivity for Human Health

**DOI:** 10.3390/molecules26216387

**Published:** 2021-10-22

**Authors:** José Nabor Haro-González, Gustavo Adolfo Castillo-Herrera, Moisés Martínez-Velázquez, Hugo Espinosa-Andrews

**Affiliations:** 1 Food Technology Unit, Center for Research and Assistance in Technology and Design of the State of Jalisco, A.C., Camino Arenero # 1227, Col. El Bajío del Arenal, Zapopan 45019, Mexico; jose_nabor_94@hotmail.com (J.N.H.-G.); gcastillo@ciatej.mx (G.A.C.-H.); 2 Medical and Pharmaceutical Biotechnology Unit, Center for Research and Assistance in Technology and Design of the State of Jalisco, A.C., Av. Normalistas 800, Col. Colinas de la Normal, Guadalajara 44270, Mexico; mmartinez@ciatej.mx

**Keywords:** clove essential oil, biological activity, chemical composition, extraction

## Abstract

Clove (*Syzygium aromaticum* L. Myrtaceae) is an aromatic plant widely cultivated in tropical and subtropical countries, rich in volatile compounds and antioxidants such as eugenol, β-caryophyllene, and α-humulene. Clove essential oil has received considerable interest due to its wide application in the perfume, cosmetic, health, medical, flavoring, and food industries. Clove essential oil has biological activity relevant to human health, including antimicrobial, antioxidant, and insecticidal activity. The impacts of the extraction method (hydrodistillation, steam distillation, ultrasound-assisted extraction, microwave-assisted extraction, cold pressing, and supercritical fluid extraction) on the concentration of the main volatile compounds in clove essential oil and organic clove extracts are shown. Eugenol is the major compound, accounting for at least 50%. The remaining 10–40% consists of eugenyl acetate, β-caryophyllene, and α-humulene. The main biological activities reported are summarized. Furthermore, the main applications in clove essential oil in the food industry are presented. This review presents new biological applications beneficial for human health, such as anti-inflammatory, analgesic, anesthetic, antinociceptive, and anticancer activity. This review aims to describe the effects of different methods of extracting clove essential oil on its chemical composition and food applications and the biological activities of interest to human health.

## 1. Introduction

According to the European Pharmacopoeia, an essential oil is an odorous product, usually of complex composition, obtained from a botanically defined raw vegetable material by hydrodistillation, steam distillation, or a suitable mechanical process [1]. Essential oils (EOs) are complex mixtures of secondary metabolites of aromatic plants [2,3]. EOs are liquid, soluble in organic solvents and soluble in lipids, some of them are colorless and others range from a light yellow to a reddish-orange, such as lemongrass oil, cinnamon oil, and sandal oil; mainly, EOs are less dense than water, such as citronella oil, lime oil or orange oil, but there are some heavier than water, such as allspice oil, cinnamon oil, clove oil or garlic oil. It is estimated that of the 3000 EOs known, only 10% are used commercially. EOs are recognized for several biological activities (bactericidal, antiviral, and fungicidal) and medicinal and aromatic properties. Among their multiple uses, they are considered suitable substances to replace chemical additives for food preservation. They also serve as an antimicrobial, analgesic, sedative, and anti-inflammatory drugs, spasmolytic agents, and local anesthetics [2,3,4]. In addition, EOs and their components are used to produce perfumes, makeup, health, dental, and agricultural products, and alternative therapies. [2].

EOs are obtained from different plant organs. The most widely used are flowers (*Jasminum* spp., *Rosa* spp., *Viola* spp., *Lavandula* spp., *S. aromaticum* L.), leaves (*Thymus vulgaris*, *Eucalyptus* spp., *Lippia graveolens*, *Ocimum basilicum*, *Salvia rosmarinus*, *Cymbopogon citratus*, *Melaleuca alternifolia*), fruits (*Illicium verum*, *Citrus sinensis*, *Citrus limon*), seeds (*Elettaria cardamomum*, *Coffea arabica*, *Piper nigrum* L.), bark (*Cinnamomum* spp.), and roots [2,5]. EOs are very complex natural mixtures that can contain more than 20 components at different concentrations. Terpenes, terpenoids, and aromatic and aliphatic components are the main constituents. The main components constitute 20–70% of the total concentration, while the rest comprises the minority components [2,3]. The relative concentration of these principal compounds determines the biological properties of EOs [2,3].

Various authors have reported that the composition and extracted yield of EOs depends on the species, soil composition, plant organ (aerial parts, inflorescence, or roots), age, cycle stage, selected extraction method, and conditions of extraction [2,5,6,7,8,9,10,11,12,13,14,15,16,17]. Mohamed et al. [16] reported that the application of different fertilizers (organic or chemical fertilizers) influences not only the growth or productivity of the plant but also the final composition and yield of the EO. Recent studies, such as the one reported by Gioffrè et al. [17], show that year after year, the composition of EO can change due to many factors, such as the geographic area of cultivation (microclimate), the agronomic techniques (fertilization, irrigation), and the date of harvest. Alfikri et al. [9] reported that the phenological stage influences the yield and quality of clove essential oil. In addition, they reported that clove flower buds in the flowering stage had the highest yield, eugenol content, and refractive index. Likewise, they report differences in the oils between young and mature trees. The best quality of clove essential oil was obtained from the buds of mature trees, while the CEO obtained from young trees had the strongest antioxidant activity [9]. Research by Hu et al. [6] notes that the storage conditions also impact the composition of EO, mainly due to the duration, temperature, and relative humidity of the storage, causing the generation or degradation of certain components [6,18]. This review focuses on differences in the chemical composition of clove essential oil (CEO) obtained through different extraction methods and the main bioactivities of interest to human health and food applications.

## 2. Clove Essential Oil (CEO)

*Syzygium aromaticum* L. belong to the *Myrtaceae* family, which has more than 3000 species and 130–150 genera, such as the myrtle, eucalyptus, clove, and guava families. Clove is an aromatic flower cultivated in Madagascar, Sri Lanka, Indonesia, and China [10,11,19]. Several reports suggest that *S. aromaticum* L. contains approximately 15–20% wt. of EO. CEO contains a high amount of phenolic compounds with several biological activities, including antibacterial, antifungal, insecticidal, and antioxidant properties [4,10,11,19,20]. The FDA classifies CEO as generally recognized as safe (GRAS); for this reason, it is used in perfumes, cosmetics, sanitary products, medicines, and foods [12,19].

### 2.1. CEO Composition

**Figure 1:**
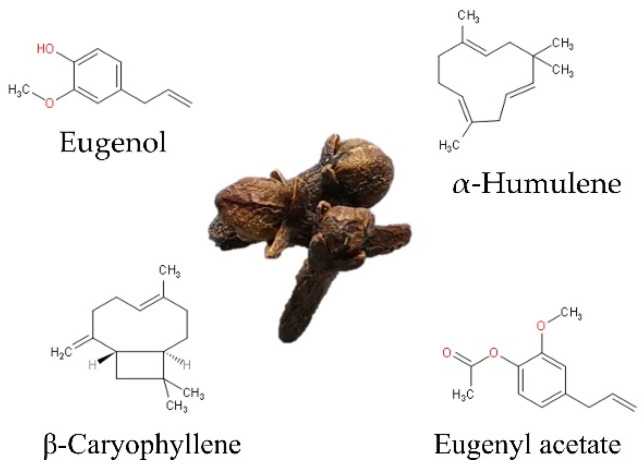
Chemical structure of main compounds of clove (*S. aromaticum* L.) essential oil.

At least 30 compounds have been identified in CEO [19]; eugenol is the major compound, accounting for at least 50%. The remaining 10–40% is made up of eugenyl acetate, β-caryophyllene, and α-humulene (Figure 1). Less than 10% correspond to minor or trace components such as diethyl phthalate, caryophyllene oxide, cadinene, α-copaene, 4-(2-propenyl)-phenol, chavicol, and α-cubebene, among others, as shown in Table 1, which shows the general composition reported by different authors [2,3,4].

#### 2.1.1. Eugenol

Eugenol is a phenylpropanoid compound found in *S. aromaticum* L., *Cinnamomum* spp., *P. nigrum*, *Zingiber officinale*, *Origanum vulgare*, and *T. vulgaris* [23]. Eugenol is a volatile compound that varies from colorless to light yellow and has low water solubility (approximately 2460 mg/L at 25 °C), a strong odor, and an intense flavor. Among the reported biological activities of eugenol are insecticidal, antimicrobial, anti-inflammatory, wound healing, antiviral, antioxidant, and anticancer activity [13,14,23,24,25,26,27].

Banerjee and coworkers [27] observed the anti-inflammatory and wound healing ability of a clove oil emulsion in murine experiments. Eugenol-treated skin showed re-epithelialization 20 days after the wound. This result was similar to that of a diclofenac gel and a neomycin cream currently used to control inflammation and heal wounds [27]. Other research reported that eugenol did not modify interleukin 8 (IL-8) levels in human skin cells (HaCat) but instead targeted other pro-inflammatory cytokines [27]. The inhibition of voltage-gated Na^+^ channels modulate the analgesic effects of eugenol. Eugenol induces the activation of transient receptor potential cation channel V1 (TRPV_1_), an effect similar to local anesthetics such as lidocaine [28].

Eugenol has shown potential anticancer activity against colon, gastric, breast, prostate, and skin cancer, as well as melanoma and leukemia [23]. Eugenol inhibits tumor proliferation and formation, increases reactive oxygen species (ROS), generates apoptosis, and has a genotoxic effect in different cancer cells [23,29,30,31]. El-Saber Batiha, G. et al. [4] collected information related to Eugenol Pharmacokinetic and Toxicity Studies. They reported that eugenol reached plasma and blood in a half-life of 14–18 h. It also showed a cumulative effect in the treatment of neuropathic pain. Although the Food and Drug Administration (FDA) has confirmed the safety of CEO as a dietary supplement, much attention has recently been paid to its toxicity due to cytotoxic activity against human fibroblasts and endothelial cells. They also reported that eugenol showed a spermicidal effect in vitro and allergic efficacy when used in dentistry [4].

#### 2.1.2. Eugenyl Acetate

Eugenyl acetate is a phenylpropanoid derivative of eugenol that exhibits antibacterial, anticancer, antimutagenic, antioxidant, and anti-virulence activity [11,19,27,32,33]. It showed inhibition of 94.5, 92.1, and 100% at 200 μg/mL against *Fusarium moniliforme*, *Harpophora oryzae*, and *Rhizoctonia solani*, respectively [34]. Eugenyl acetate has been described as a potent antioxidant agent; it showed 90.30% DPPH free radical scavenging at 35 µg/mL, and 89.30% NO free radical scavenging at 60 µg/mL. It also exhibited potential antifungal activity against *Candida* spp. and inhibited biofilm formation capacity [33]. Pasay et al. (2010) reported high toxicity against human scabies mites [32]. Eugenyl acetate also showed 100% toxicity against *Artemia salina* at 0.3 μg/mL. The low lethal concentrations obtained for eugenyl acetate could also indicate toxicity to other organisms, such as disease vector insect larvae [32]. Eugenyl acetate had an LC_50_ of 0.1 mg/mL against *Aedes aegypti*, showing potential utility as a larvicide [35]. The larvicidal action is mainly due to interference with the octopaminergic system [36]. The antioxidant, antimicrobial, antitumor, and larvicidal properties have increased its demand in the food and cosmetic industries [11,19].

##### 2.1.3. β-Caryophyllene

β-Caryophyllene is a sesquiterpene found in clove (*S. aromaticum* L.), hemp (*Cannabis sativa* L.), black pepper (*P. nigrum* L.), *Eugenia cuspidifolia*, *Eugenia tapacumensis*, and guava leaves (*Psidium cattleianum Sabine*) [37,38]. β-Caryophyllene is insoluble in water but is soluble in ethanol. It has demonstrated antimicrobial, anticarcinogenic, anti-inflammatory, antioxidant, anxiolytic-like, and local anesthetic effects and anticancer properties, including against prostate, breast, pancreatic, skin, leukemia, lymphatic, and cervical cancer [11,14,21,37,38,39,40,41]. These studies suggest that β-caryophyllene decreases cell growth and proliferation in colon cancer, interfering with the stages of tumor development and reducing the activity of extracellular matrix metalloproteinases. β-Caryophyllene can act as a chemosensitizer, improving the effectiveness of drugs against tumor cells [37,38,40]. It is also effective against *Anopheles subpictus* (LC_50_ = 41.66 μg/mL), *Aedes albopictus* (LC_50_ = 44.77 μg/mL), and *Culex tritaeniorhynchus* (LC_50_ = 48.17 μg/mL). Dahham et al. [41] reported that the radical scavenging ability of β-caryophyllene was approximately 1.25 and 3.23 μM by the DPPH and FRAP scavenging methods, respectively. These results indicate that β-caryophyllene has high antioxidant activity.

##### 2.1.4. α-Humulene

α-Humulene is a sesquiterpene found in *S. aromaticum* L., *Senecio brasiliensis*, *Humulus lupulus* L., and *Salvia officinalis* L. This compound has shown anti-inflammatory and antitumor activity in lung, colon, prostate, and breast cancer. Some studies reported that α-humulene demonstrated antiproliferative activity and alteration of the mitochondrial cell membrane in colon cancer cells [14,37,42,43,44,45,46]. It can also improve the antiproliferative effect of cytostatic drugs and other anticancer bioactivities [42,44]. Nguyen et al. [46] reported that α-humulene inhibits the activity of the CYP3A enzyme, a drug-metabolizing enzyme in humans’ and rats’ liver microsomes [46]. Fernandes et al. [47] reported that oral treatment with α-humulene and β-caryophyllene (50 mg/kg) produced comparable anti-inflammatory effects with dexamethasone treatment in model mice and rats. α-Humulene prevents the generation of TNFα, while β-caryophyllene only decreases its release. In addition, they reduce the production of prostaglandin E_2_, the inducible expression of nitric oxide synthase, and cyclooxygenase. α-Humulene exhibited larvicidal activity against three vector mosquitoes, *An. Subpictus* (LC_50_ = 10.26 μg/mL), *Ae. albopictus* (LC_50_ = 11.15 μg/mL), and *Cx. tritaeniorhynchus* (LC_50_ = 12.05 μg/mL) but was shown to be safe for *Gambusia affinis* (LC_50_ = 1024.95 μg/mL). It showed larvicidal LC_50_ of 20.86 µg/mL and EC_50_ of 77.10 µg/mL on *Helicoverpa armigera* eggs. α-Humulene has also been evaluated against beetle species that attack stored products [35,48,49]. The toxicity of α-humulene against *Sitophilus granarius* was LC_50_ = 4.61 µL/mL, and it reduced the respiration rate of *S. granarius* at 1 and 3 h after exposure [50].

## 3. Extraction of EOs

**Figure 2:**
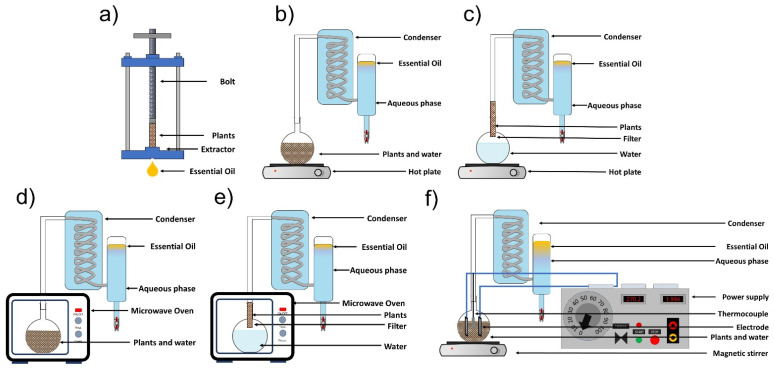
Essential oil extraction methods: (**a**) cold pressing, (**b**) hydrodistillation, (**c**) steam distillation, (**d**) microwave-assisted hydrodistillation, (**e**) microwave-assisted steam hydrodistillation, and (**f**) ohmic heating-assisted hydrodistillation (adapted from Golmakani [19] and Hatami et al. [20]).

EOs are extracted from plant feedstock by conventional methods, including cold pressing, hydro-distillation, and steam distillation. Additionally, we can include innovative techniques such as microwave-assisted hydrodistillation, microwave-assisted steam distillation, and hydrodistillation assisted by ohmic heating (Figure 2).

**Figure 3:**
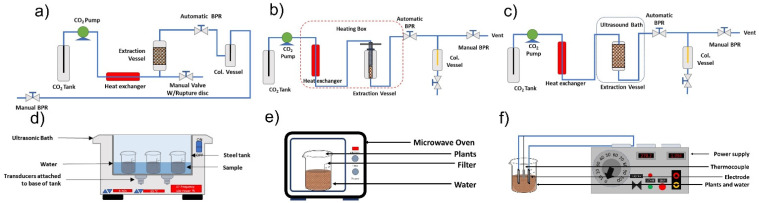
Organic extract extraction methods: (**a**) supercritical fluid extraction, (**b**) cold pressing-assisted supercritical fluid extraction, (**c**) ultrasound-assisted supercritical fluid extraction, (**d**) ultrasound-assisted extraction, (**e**) microwave-assisted extraction, and (**f**) ohmic heating-assisted extraction (Hatami et al. [20] and Fragoso-Jiménez et al. [51]).

In addition, the methods used for organic extracts including ultrasound-assisted extraction (UAE), solvent extraction (SO) and supercritical fluid extraction (SFE), microwave-assisted extraction, ohmic heating-assisted extraction (Figure 3). These extracts have a volatile fraction, which is sometimes erroneously called essential oil [5,10,11].

### 3.1. Conventional/Classical Extraction Methods

The conventional extraction methods are based on the distillation process by heating a plant matrix to recover EO [5]. The extraction is done by injecting steam or water, which crosses the plant matter from the bottom up and carries the volatile materials together with the water as if they were a single component. EO is immiscible in water, making it easily removable by decanting [5]. The HD and SD methods are the most extensively used for extracting EOs. These are easy to operate, have high reproducibility, and do not use organic solvents [11,19,52]. However, these methods have several drawbacks, including a long extraction time, the use of large volumes of solvent and energy, and possible thermal degradation and hydrolysis of some of the components of interest from prolonged contact with boiling water or steam. However, these compounds resulting from hydrolysis belong to the final composition of EO [5]. It is important to note that the conventional methods of extracting essential oils have few adjustable parameters that control the selectivity of the processes and the final concentration of the essential oil [14,19].

### 3.2. Advanced/Innovative Extraction Methods

Advanced extraction methods, including microwave-assisted extraction (MAE), ultrasonic-assisted extraction (UAE), subcritical fluid extraction, and supercritical fluid extraction (SFE), improve extraction performance, reduce extraction time and energy consumption to obtain organic extracts [3,8,10]. These methods improve organic compound extraction yield by applying microwave or ultrasonic energy, which can destroy the cell walls of the plant matrix, allowing the compounds to flow better from the biological material [5,15,21]. Kennouche et al. [21] reported that the essential oil of *Eugenia caryophyllata* seed obtained by MAE and MSD contained a high percentage of eugenol (65–71%). These extracts preserved their antimicrobial and antioxidant properties. MAE reduces energy consumption, heating time, and organic extract degradation [19,21,22]. Ultrasonic waves from 20 to 100 kHz can be applied by direct contact with the sample (ultrasound system coupled with a probe) or indirectly through the walls of the sample container (ultrasonic bath). Acoustic power and wave frequencies applied in liquid media can produce the acoustic cavitation phenomenon, where the creation, expansion, and implosion of bubbles enhance the selectivity of target molecules [12,53,54].

SFE is used to selectively remove chemical compounds using a solvent in its supercritical state, typically carbon dioxide [55]. Additionally, co-solvents such as methanol, ethanol, or water change the density, viscosity, and solvation power of the supercritical solvent, promoting the extraction of specific compounds [5,14]. The SFE process reduces undesirable organic pollutants, toxins, and pesticide residues present in the biological material [5].

### 3.3. Effect of the Extraction Method on the Concentration of the Main Volatile Compounds of the Essential Oil and Organic Extract

It is well known that differences in the composition of EOs and organic extract depend on the species [7], the phenological stage [9], agroecological conditions [6,8,16,17], pretreatment [11,14], processing conditions [6,10,11,12,13,14,18], and extraction method [10,11,12,13,14]. Table 2 summarizes the impact of the different extraction methods and conditions on the concentration of the main volatile compounds in cloves.

The main volatile compounds obtained by the different extraction methods were similar; however, the concentration of each compound was different. The CEO obtained by the different processes was a characteristic pale yellow color. However, soxhlet extraction (SO) using ethanol could produce a brown organic extract due to impurities, waxes, and organic waste [11]. Golmakani et al. [19] reported that the extraction yield from MA HD after 60 min was similar to the final yield from HD after 240 min. Similarly, MA SD operated almost 4.8 times faster than SD [19]. The MAE reduces the extraction time from 10 to 2 h and generates an increased extraction yield by two-fold, with less severe parameters applied [11,12,14,19]. This occurs because these methods allow the extraction temperature to be reached shorter than conventional methods. However, parameters must be carefully controlled because exposure can alter the chemical composition of EOs [11,12,14,19].

Tekin et al. [54] reported that organic extract obtained by UAE (53 kHz) had significant eugenol, α-Caryophyllene, and eugenyl acetate contents [54]. Ghule and Desai [57] used ultrasound-assisted hydrotropic extraction to isolate eugenol and eugenyl acetate from clove buds. The extraction yield was approximately 20% applying 158 W sonication power (26 kHz with a 7 mm diameter probe) to 8.2 g ground clove buds in 150 mL of sodium cumene sulfonate 1.04 M for 30 min at 38 °C [57].

The main factors in the extraction of the organic extract by SFE are particle size, temperature, pressure, and extraction time. Extraction yield increases by decreasing the crushed particle size of the clove because the diffusion paths are shorter and result in less resistance to diffusion between particles. The temperature and pressure of extraction modify the CO_2_ density; thus, the extraction yield is higher due to the increased solubility of clove components. However, there is a risk that high-molecular-weight compounds (fatty acids, fatty acid methyl esters, sterols, etc.) may also be extracted in the organic extract [11,12,14].SFE offers substantial advantages over other methods in the extraction of organic extracts, including higher extraction performance, a higher percentage of active antioxidant ingredients, shorter extraction time, etc. [11,12,14].

However, SFE requires expensive equipment, highly trained operators, and high operating and maintenance costs. Likewise, the incorporation of a co-solvent, continuous operation, and CO_2_ recycling could increase the cost of separation without considering the impact on the environment [14,20,55]. Hatami et al. [20] conducted an economic evaluation of obtaining a clove extract. They considered a price of USD 40.00/kg of extracted CEO, obtaining a possible annual income equivalent to USD 5.9 million, in addition to a 79% gross margin for every dollar invested, with the time for recovery of the initial investment ranging between 4 and 14 months [20].

## 4. Food Applications

In recent years, food industries have faced great challenges in producing safe foods with a longer shelf life while preserving nutritional value and sensory characteristics. The deterioration of food causes significant economic losses. It is also harmful to human health due to the toxic secondary metabolites produced. Growing consumer demand for natural alternatives to synthetic preservatives in food has made EOs a natural substitute due to their antioxidant, antibacterial, and antifungal properties [58,59,60,61,62,63]. However, the main challenge of EOs as food preservatives lies in maintaining their functional properties without changing the taste of food and increasing consumers’ appetite (Table 3) [58,63].

Generally, complex food matrices require higher EO concentrations than those used in in vitro tests. For example, foods with high protein content can produce protein–phenolic EO complexes, reducing the effectiveness of EOs [58,59,60,64]. Further, the lipid fraction of food can absorb the antimicrobial agent, reducing its bactericidal action. Likewise, reducing the water content in food could hinder the transfer of antimicrobial agents to the active site in the microbial cell [58,59,60,64]. Furthermore, external factors such as storage temperature, packaging, initial concentration, application method, and the type of microorganism can interfere with the effectiveness of the EO [59,65].

### 4.1. Baked Food

The baked food industry emphasizes the prevention of mold growth and the maintenance of safety and nutrition [58,65,66]. The preservative methods for baked foods include modified storage atmosphere, irradiation, aseptic packaging, and preservative acids. However, the use of organic acids (propionic, benzoic, and sorbic acids) has been restricted in several countries due to their negative impact on human health [58,65,66]. Eugenol provides CEO with broad-spectrum activity against deterioration and foodborne pathogenic microorganisms such as *Penicillium* spp., *Aspergillus* spp., *Escherichia coli*, and *Staphylococcus aureus*. Adding it to baked foods can extend the shelf life without affecting the original taste, flavor, texture, appearance, or sensory acceptability [58,65,66].

### 4.2. Dairy Products

Consumption of dairy products such as cheese has been responsible for various outbreaks of foodborne diseases [59,71]. Ahmed et al. [59] applied approximately 1 kg of CEO per 200 liters of raw milk as an antimicrobial agent for cheese production. The CEO demonstrated significant antimicrobial action without affecting organoleptic properties during 1 month at 4 °C, showing a potential cost-effective use [59].

### 4.3. Processed Food

In recent years, the market for ready-to-cook processed foods (pre-cooked foods) has expanded due to lifestyle changes and the development of refrigerated distribution networks. The microbial deterioration of processed foods causes unpleasant odors, discoloration, stickiness, sediments, gases, and decreased pH, reducing the quality of food products and putting consumers’ health at risk. The addition of 5% (*w*/*w*) CEO to processed foods has been shown to have a negative effect on the organoleptic properties of foods, so the focus of its application has been on its use as a flavoring component with antimicrobial and antioxidant properties [62,63,64].

### 4.4. Meat, Poultry, and Seafood Products

The application of CEO to animal food products reduces undesirable reactions involving the deterioration of taste, smell, color, texture, and sensory properties [60,63,67,68,71,73]. Its antimicrobial activity generates a decreased bacterial count, decreases the deamination capacity of non-protein nitrogenous compounds, and reduces hydroperoxide formation due to its antioxidant properties. Mechanisms behind the antioxidant properties of CEO include transition metal binding, inhibition of chain reactions, breakdown of hydroperoxides, and interaction with free radicals. CEO has been applied to white shrimp, salmon burgers, fish fillets, ground beef, chicken patties, and chicken breast meat during refrigerated or frozen storage [60,63,67,70,73,74,75]. Films fortified with CEO can reduce the loss of weight, water activity, lipid oxidation, color change, and microorganism growth in foods of animal origin for up to 45 days if heat-treated and 12 days if refrigerated [61,68,70].

### 4.5. Vegetables

Post-harvest vegetable deterioration during transport and storage leads to significant economic losses along the supply chain [83,84,86]. The antimicrobial properties of CEO can prevent fungal spoilage in vegetables and adverse health effects, and it can serve as a potential alternative to chemical fungicides. The antimicrobial activity can be improved when it is combined with UV-C light treatment or modified packaging. These processes allow effective control of post-harvest decomposition and preserve the physicochemical quality of vegetables, prolonging their useful life without affecting organoleptic properties [83,84,85,86]. CEO is added to the wash treatment of fresh-cut vegetables as an alternative to acetic acid, sodium bicarbonate, and chlorine-based disinfectants, reducing microbiological hazards and extending the shelf life. Additionally, CEO wash does not impact color attributes, bioactive content, composition, or sensory attributes. Therefore, CEO application in conjunction with cold storage is an excellent ecological substitute that could be further improved for commercial applications to bring vegetables to market with better and longer-lasting post-harvest quality with greater consumer acceptance [85,86].

### 4.6. Packaging Materials

Recently, new biodegradable packaging materials have been developed from natural polymers (polysaccharides, lipids, proteins). Their antioxidant and antimicrobial properties can be enhanced by incorporating essential oils to extend the shelf life and reduce or inhibit foodborne pathogens [71,80]. The incorporation of EO in films for coating is aimed at modifying the functional properties, such as water vapor permeability and antimicrobial and antioxidant properties [68,77,78,79,81]. CEO-fortified films showed antibacterial properties and growth inactivation for up to 21 days due to the penetration and destruction of the cell structure by CEO compounds [79]. The addition of CEO can modify the moisture content of packaging materials, improving the spatial distance within the film matrix, resulting in thicker films [77,78,79,80,81].

The optical properties of films affect the appearance and quality of foods. The application of a coating also reduces the rate of lipid oxidation [77,81]. In this regard, the different coloring components of CEO can change the color of the films [77,81]. Its incorporation increases opacity values due to an increase in light scattering caused by oil droplets in the film network. These reduce transparency, which represents an advantage for photosensitive food [77].

The incorporation of CEO in a film network partially replaces the stronger polymer–polymer interactions with weaker interactions (polymer–oil). This generates a more heterogeneous network and a discontinuous microstructure by a rearrangement of the polymers. Likewise, the incorporation of CEO has a plasticizing effect, decreasing the glass transition temperature and the elastic modulus of films [77,78,79,80,81]. Sarıcaoglu and Turhan [77] observed a decrease in elastic modulus and tensile strength when CEO was added to films made from mechanically deboned chicken meat protein. However, the tensile strength was kept above 3.5 MPa, a recommended value for coating film on food [77,79,80,81]. These changes in the structure due to the incorporation of CEO also produced rougher and more porous films [77,78,81].

## 5. Biological Activities of CEO

CEO has been shown to have different health benefits, mainly due to the eugenol content. However, the other compounds have various health benefits too. The principal biological activities of CEO are shown in Table 4 [4,87].

### 5.1. Antimicrobial

CEO has shown broad-spectrum inhibitory activity against pathogens. The antibacterial mechanism has been related to the -OH groups located at the meta and ortho positions, respectively, in the main chemical composition. These functional groups can interact with the cytoplasmic membrane of microbial cells [92,96,97,99,100]. CEO can permeate through the cell membrane due to its lipophilic properties. The interaction of CEO with polysaccharides, fatty acids, and phospholipids causes loss of cellular membrane integrity, leakage of cellular contents, and interference with proton pump activity, leading to cell death [92,96,97,100,110]. CEO can inhibit Gram-negative bacteria (*E. coli*, *Salmonella*, *Klebsiella pneumoniae*, *Erwinia carotovora*, *Agrobacterium*, and *Pseudomonas aeruginosa*) and Gram-positive bacteria (*S. aureus*, *Streptococcus*, and *L. monocytogenes*), *Aspergillus* (*A. flavus*, *A. parasiticus*, and *A. ochraceus*), *Penicillium*, *C. albicans*, and yeast [92,96,97,100]. CEO inhibits Gram-positive bacteria to a greater extent than Gram-negative bacteria. This is attributed to a diffusible mucopeptide layer in Gram-positive bacteria that makes them susceptible to antimicrobial agents. In contrast, the complex layer of lipopolysaccharide in the outer cell membrane of Gram-negative bacteria can significantly reduce the diffusion rate of lipophilic antibacterial compounds through the cell membrane [97]. Likewise, food-related pathogens have shown greater sensitivity to CEO than probiotics and fungi [100].

### 5.2. Antioxidant

CEO has the antioxidant compounds eugenol, eugenyl acetate, β-caryophyllene, and α-humulene, which protect cells from free radical oxidation. Diseases such as cancer, arteriosclerosis, Alzheimer’s disease, and Parkinson’s disease are related to the presence of ROS compounds [110]. CEO has shown scavenging activity on radicals and inhibition of lipid peroxidation [41,93]. The hydroxyl group available in eugenol on the aromatic ring is responsible for the antioxidant activity [99]. The phenolic compounds transfer electrons or hydrogen atoms and neutralize them to free radicals, resulting in a blocked oxidative process [100].

CEO has a protective effect on biochemical changes and histopathological injuries in the kidney, liver, and brain induced by ROS. The main ROS changes inhibited were increased lipid parameters (HDL-C, TC, LDL-C, and VLDL), blood electrolyte (Na^+^, K^+^, and Cl^−^) and creatinine levels in the liver, hepatic enzymes, blood urea, increased liver and kidney weight, increased serum creatinine, and decreased total protein and albumin [102]. Marmouzi et al. [99] reported that CEO antioxidant activity in three test methods was 150 mg TE/g EO for DPPH, 110 mg TE/g EO for ABTS^+^, and 34 mg AAE/g EO for FRAP.

### 5.3. Insecticidal

Insect-borne diseases are an ongoing challenge to public health. Some species are invasive urban pests, transmitting numerous pathogenic microorganisms and causing allergic reactions and asthma in young and older people. Commonly used insecticides cause significant health problems and have long-lasting adverse effects on the environment. Moreover, an increase in resistance against insecticides has been reported. Due to this, investigations have focused on developing natural insecticides based on EOs to control agricultural and urban pests [105,106,107]. However, their high volatility decreases the time during which EOs remain in the human body, so sometimes several applications are required in a day.

CEO has shown high levels of repellency and fumigant toxicity on flea, aphids, nymphal instars, mites, imported red fire ants, *C. pipiens*, and American and German cockroaches [103,105,106]. The oviposition-deterrent activity of CEO can be found in other mosquito species (*Anopheles stephensi*, *An. subpictus*, *Ae. aegypti*, *C. pipiens*, *Ae. albopictus*, *Culex quinquefasciatus*, and *Cx. tritaeniorhynchus*) [105,107]. It targets the egg stage as an oviposition deterrent and the larval stage as a larvicide against *Ae. japonicus*, *Ae. aegypti*, and *Cx. quinquefasciatus*. CEO has shown repellent action in the laboratory and field settings against adult *Ae. aegypti*, *Ae. cinereus*, and *Ae. communis* [105,107].

The primary targets of CEO and other EOs are octopamine and gamma-aminobutyric acid (GABA) receptors and transient receptor potential (TRP) channels [104]. The dose–response ratio of CEO showed an increased mortality rate with increasing concentration [105]. CEO increased permeability activity on the cell membrane, disrupted the cytoplasmic membrane, and interacted with proteins, ATPase, histidine decarboxylase, amylase, and protease enzymes, which were also inhibited.

Lambert et al. [103] evaluated the activity of CEO against adult *C. felis felis* and the development of their eggs. The LC_50_ was 5.70 μg/cm^2^ against adult fleas and 0.30 g/cm^2^ against flea eggs; however, the insecticidal activity of eugenol was three times higher [103]. Toledo et al. [104] reported that it had activity against aphids, but not against ladybugs. They reported an LC_95_ of 0.17 μL/cm^2^ for aphids, while the same dose only had a lethality of less than 18% for *Corymbia maculata*. The ladybugs that were exposed to CEO did not exhibit impaired locomotion ability. Therefore, it was concluded that the application of CEO represents an alternative to control aphid infestations [104]. Elzayyat et al. [105] evaluated the insecticidal activity against adults and larvae of *Culex pipiens*, and reported an LC_50_ of 0.374 and 0.036%, respectively [105]. Neupane et al. [106] observed that CEO, eugenol, and eugenyl acetate applied at 4.0 mL/cm^2^ provided 95, 85, and 87% mortality of German cockroaches, respectively. They also reported repellency for 30 min by applying 80% CEO. Reuss et al. [107] observed that CEO functions as an oviposition repellent and a larvicide, with an LC_50_ of 17 mg/L.

CEO and its main constituents are products that have low toxicity to mammals and zero residual concentration. Its application is limited to plague insect control, which is essential to prevent infestations in the environment [103].

### 5.4. Antiviral

CEO has shown antiviral activity against Ebola [111], influenza A virus [112], and herpes simplex virus types 1 and 2 [111]. Recent studies by de Oliveira et al. showed that eugenol derivatives could inhibit the activity of the West Nile Virus, providing a promising compound against flaviviruses such as dengue, Zika, and yellow fever [24]. Eugenol has also been studied as a possible inhibitor of the initial stage of HIV-1 infection because it can reduce virus replication. Likewise, eugenol can increase lymphocyte production; therefore, the lymphocyte proliferation capacity of eugenol may be responsible for its anti-HIV-1 activity [25].

CEO has demonstrated antiviral activity against feline calicivirus, which is used as a substitute for human norovirus. For this reason, the application of CEO in the process of washing fruits and vegetables eliminates any viral load that may exist. In addition, the application of CEO in cleaning wipes allows the decontamination of surfaces [113]. Furthermore, CEO has been shown to increase the resistance of tomato plants to tomato yellow leaf curl virus more than moroxydine hydrochloride [114].

### 5.5. Antinociceptive

Nonsteroidal anti-inflammatory drugs (NSAIDs) are the most widely used drugs to treat inflammatory nociceptive pain. Their principal mechanism is cyclooxygenase (COX) inhibition, decreasing the prostaglandins that cause nociceptive pain. The antinociceptive and anti-inflammatory activities of eugenol are related to COX-2 inhibition and vanilloid transient receptor potential (TRPV) by high-voltage Ca^2+^ current inhibition in primary afferent neurons [101]. This antinociceptive response is related to opioid, cholinergic, and *α*2-adrenergic receptors, but not serotoninergic receptors. The antinociceptive effect of eugenol is probably related to gamma-aminobutyric acid (GABA) receptor modulation, because eugenol administration inhibits GABA receptor currents in trigeminal ganglion neurons and inhibits GABA *α*1*β*2γ2 expressed in these neurons [28,101].

### 5.6. Anti-Inflammatory and Wound Healing

Oxidative stress and inflammation are near-related processes in many pathophysiological conditions such as diabetes, hypertension, and cardiovascular and neurodegenerative diseases [99]. The anti-inflammatory properties of CEO and eugenol are comparable to diclofenac gel, reducing inflammation by 60 to 20% after 3 h. Likewise, induced wounds in rats treated with CEO showed a significant contraction of more than 95% in the first 15 days. These results demonstrate that animals treated with CEO underwent similar healing to those treated with neomycin, which is currently used to control inflammation and heal wounds. Therefore, the chronic and acute side effects of synthetic antibiotics can be avoided, especially if they are given frequently [27]. CEO inhibited important antiproliferative biomarkers whose activity depends on their concentration. It decreased the levels of inflammatory biomarkers such as VCAM-1, IP-10, I-TAC, and MIG, in addition to inhibiting the tissue remodeling protein molecules collagen I, collagen III, M-CSF, and TIMP-1 [26,87,109]. The application of CEO can reduce epidermal thickness and the number of inflammatory cells expressing COX-2 without affecting COX-1. The mechanism of eugenol, as an anti-inflammatory, inhibits the expression of COX-2 and reduces the production mediators of inflammation [26,87]. Eugenol has also been reported to not alter IL-8 levels in human skin keratinocytes but to target other pro-inflammatory cytokines in pre-inflamed human dermal cells [27]. These results suggest that CEO possesses anti-inflammatory activity and favors wound healing.

### 5.7. Analgesic

Headaches, joint pain, toothaches, and oral hygiene issues have traditionally been treated with aromatherapy and CEO. The CEO and eugenol are safe, effective, and inexpensive analgesics, and the analgesic effect of eugenol in different pain models has been well documented [28]. Khalilzadeh et al. [28] reported that the analgesic effect of CEO is mediated by the opioidergic and cholinergic systems. The analgesia produced by CEO in acute corneal pain appears to depend on the cholinergic activity. The analgesic and local anesthetic effects of eugenol can be modulated by its inhibitory effect on voltage-gated channels (Na^+^ and Ca^2+^) and activation of TRPV1. The analgesic effects of CEO and eugenol are very similar to those of lidocaine. Correia et al. [88] demonstrated the analgesic efficacy of CEO in fish. When it was used in concentrations between 40 and 80 µL/L in procedures that were invasive or could cause pain, an analgesic effect in animals was reported, minimizing the effects of harmful stimuli. CEO has potential for use in painful procedures, to minimize the effects of harmful stimuli for ethical reasons, and to ensure the welfare of the animal, avoiding stress and its negative consequences [88,105,115].

### 5.8. Anesthetic

CEO is recognized as an anesthetic at low concentrations (50–500 µL/L) in vertebrates and invertebrates without side effects. It induces anesthesia faster, has brief reflex recovery, and shows a low mortality rate without affecting external stimulus response [88,90,91]. Recent studies showed that topical application of CEO and eugenol reduces corneal sensitivity in rats similar to lidocaine [28]. The maximum level and duration of anesthesia depending on the concentration and time of exposure, which differs between chemicals. CEO efficiently induces anesthesia in Nile tilapia, cardinal tetra, ringed cichlid, and angelfish, affecting swimming ability and balance, and decreasing the response to external stimuli until complete immobilization. Depending on the concentration of the dose, the time to achieve full anesthesia is decreased. Furthermore, there are no side effects of CEO based on the concentration and time of exposure when recovering from anesthesia [28,89,91]. CEO is an effective anesthetic for red claw crayfish and other crustaceans, including *Nephrops norvegicus* and grass shrimp. Induction and recovery times increase with increased crayfish size, as these are related to oxygen demand. Absorption and elimination of CEO are measured by the oxygen consumption rate, the relationship between the body and the gill surface, and the gill infusion rate. Size is inversely related to anesthetic efficacy [90]. For invasive and painful procedures, the use of CEO is recommended due to its better anesthetic effect [88].

### 5.9. Anticancer

The eugenol, α-humulene, and β-caryophyllene components of CEO, which have cytotoxic and antitumor activity, have been used as alternatives in the prevention and co-treatment of cancer. Some reports suggest that EOs reduce the side effects of chemotherapy, which include nausea, vomiting, loss of appetite, and weight loss [110,116,117]. The anticancer activity is mainly attributed to the antioxidant and anti-inflammatory activity, since the production of ROS specifically activates signaling pathways and contributes to the development of tumors by regulating cell proliferation, angiogenesis, and metastasis [110,116]. CEO has been tested against different cancer types, such as colon [41,93], lung [93,95], breast [41], pancreatic [41], leukemia [41,94], cervical [41], and prostate [41,93].

The anticancer properties are due to the following mechanisms: the activation of detoxifying enzymes, the destruction of DNA by oxidative stress, antimetastatic and cytotoxic activity, decreased viability, cell cycle arrest or apoptosis, the reduction of phosphate-Akt expression levels, and MMP-2 and protein leakage [87,92,95,108]. CEO has shown a low cytotoxic effect on normal cells, improving their antiproliferative activity [87,95,108].

### 5.10. Other Bioactivities

Several authors have mentioned that CEO has antiseptic [12,28,100], natural stimulant [100], carminative [11,12,100,118], anticoagulant [23,98], anthelminthic [100], antiemetic [11], antidiarrheal [12,23,98], antispasmodic [11,12], hepatoprotective [118], spasmolytic [118], antimutagenic [21,23,31], anticonvulsant [21], antidepressant [119], renal reinforcement [11,23], antipyretic [27,98], neuroprotective [23], antistress, antiallergic [11,12,28], antidiabetic [23,97], and hypocholesterolemic effects [23]. However, to our knowledge, these effects have not been completely studied, and represent new research opportunities for CEO.

## 6. Conclusions and Future Prospects

CEO is a food additive generally recognized as safe by the FDA. The chemical composition of CEO is directly affected by the phenological stage, agroecological conditions, pretreatment, processing conditions, and extraction methods. Innovative methods allow the selective extraction of bioactive compounds responsible for their health benefits. Eugenol, β-caryophyllene, α-humulene, and eugenyl acetate are the main volatile compounds with antioxidant, antimicrobial, anti-inflammatory, analgesic, antiviral, and anticancer properties. The CEO’s antioxidant and antibacterial activities have encouraged their application in meat, poultry and seafood, vegetables, dairy products, and edible coating films in the food industry.

Even though CEO is widely consumed and applied, there are still potential areas for investigation. More studies are needed to define the roles of the main components in the various biological activities for potential application in the treatment of different diseases. In addition, it is necessary to determine whether there is synergy or antagonism among these components. Likewise, it is necessary to study the application of CEO in the food industry, mainly its use as an antioxidant or antimicrobial agent without negatively affecting the color, taste, smell, and texture of foods. Few reports were found on CEO encapsulation and its effects on the main physicochemical and biological properties. More research is still required to determine the effect of encapsulation systems on solubility, absorption, bioavailability, and shelf life by avoiding degradation (photo, oxidative, or thermal) and its effect on organoleptic properties.

Despite all the studies carried out, some properties and applications have not been thoroughly investigated. Thus, opening possibilities for investigating the effect of CEO against other diseases and its future application in industries such as pharmaceuticals, foods, cosmetics, dentistry, agriculture, and others.

## Figures and Tables

**Table 1:** Comparison of chemical composition (%) of CEO reported by different authors.

		Golmakani, M.T., et al. 2017 [19]					Kennouche, A., et al. 2015 [21]			González-Rivera, J., et al. 2016 [22]	
	Compound	Retention Index	Relative Peak Area (%)				Retention Index	Relative Peak Area (%)		Retention Index	Relative Peak Area (%)
			HD	SD	MA HD	MA SD		MA SD outside	MA SD inside		Coaxial MA HD
1	Eugenol	1359	87.3	82.7	88.8	83.4	1360	65.36	71.84	1367	66.9
2	Eugenyl acetate	1526	10.4	15.6	7.46	14.3	1519	5.71	9.49	1529	2.7
3	β-Caryophyllene	1415	1.35	0.91	2.65	1.37	1446	24.62	15.6	1422	24.8
4	α-Humulene	1449	0.19	0.13	0.4	0.21	1455	-	0.01	1454	3.1
5	Caryophyllene oxide	1578	0.2	0.17	0.19	0.22	-	-	-	1367	0.1
6	α-Copaene	-	-	-	-	-	1377	0.01	tr	1378	0.8
7	Chavicol	1251	0.31	0.24	0.22	0.22	1253	0.13	0.1	-	-
8	Methyl salycilate	1191	0.08	0.08	0.1	0.07	1188	0.1	0.08	-	-
9	Benzaldehyde	956	0.07	0.08	0.06	0.05	-	-	-	-	-
10	Benzyl acetate	1161	0.05	0.04	0.06	0.05	1161	0.02	0.02	-	-
11	2-Nonanone	1089	0.04	0.04	0.05	0.04	-	-	-	-	-
12	Benzyl benzoate	1759	0.02	0.03	0.01	0.02	-	-	-	-	-
13	Ethyl benzoate	1167	0.01	0.01	0.01	0.01	1181	0.02	0.02	-	-
14	1,8-Cineole	-	-	-	-	-	1032	0.03	0.03	-	-
15	1,3,8-*p*-Menthatriene	-	-	-	-	-	1110	0.03	0.01	-	-
16	2-Heptanone	-	-	-	-	-	881	0.01	tr	-	-
17	2-Heptyl acetate	-	-	-	-	-	1043	0.03	0.01	-	-
18	2-Nonanol	-	-	-	-	-	1098	0.01	tr	-	-
19	6-Methyl coumarin	-	-	-	-	-	1549	0.03	tr	-	-
20	Acetophenone	-	-	-	-	-	1078	0.03	0.01	-	-
21	Caryophyllene alcohol	-	-	-	-	-	1565	0.04	tr	-	-
22	Epizonarene	-	-	-	-	-	1492	0.07	0.05	-	-
23	Germacrene D	-	-	-	-	-	1484	0.14	0.09	-	-
24	Methyl benzoate	-	-	-	-	-	1087	0.01	tr	-	-
25	Methyl eugenol	-	-	-	-	-	1404	0.04	tr	-	-
26	Methyl undecanoate	-	-	-	-	-	1420	0.02	tr	-	-
27	N-Citronellyl butyrate	-	-	-	-	-	1532	0.01	tr	-	-
28	Viridiflorol	-	-	-	-	-	1591	0.02	-	-	-
29	Z-Nerolidol	-	-	-	-	-	1534	0.06	0.02	-	-
30	α-Pinene	-	-	-	-	-	934	tr	0.03	-	-
31	β-Cubebene	-	-	-	-	-	1382	0.02	tr	-	-
32	β-Pinene	-	-	-	-	-	997	tr	tr	-	-
33	γ-Gurjunene	-	-	-	-	-	1470	2.35	1.65	-	-
34	δ-Cadinene	-	-	-	-	-	1500	0.22	0.2	1523	0.6
35	ρ-Acoradiene	-	-	-	-	-	1461	0.03	0.01	-	-
36	ρ-Cymene	-	-	-	-	-	1020	tr	0.07	-	-

HD, hydrodistillation; SD, steam distillation; MA, microwave-assisted.

**Table 2:** Effect of the extraction on the concentration of the main volatile compounds of cloves (%).

Method	Extraction Conditions	Extraction Product	Eugenol (%)	β-Caryophyllene (%)	α-Humulene (%)	Eugenyl Acetate (%)
HD [22]	360 min 100 °C Clove:Water 1:5	EO	87.10	5.10	0.60	6.40
HD [56]	Commercial	EO	85.50	7.40	1.50	2.7
HD [34]	240 min 100 °C Clove:Water 1:10	EO	69.68	12.23	1.50	14.38
HD [21]	150 min 100 °C Clove:Water 1:2	EO	64.91	22.01	-	6.31
HD [19]	240 min 100 °C Clove:Water 1:10	EO	87.26	1.35	0.19	10.43
HD [11]	360 min 100 °C Clove:Water 1:5	EO	58.20	20.59	2.61	13.84
Microwave-assisted HD [21]	30 min 850 W 100 °C Clove:Water 1:5	EO	69.52	17.20	0.01	9.11
Microwave-assisted HD [19]	80 min 1000 W 100 °C Clove:Water 1:10	EO	88.80	2.65	0.40	7.46
Microwave-assisted HD coaxial [22]	120 min 300 W 100 °C Clove:Water 1:5	EO	66.90	24.80	3.10	2.70
Microwave-assisted SD [19]	80 min 1000 W 100 °C Clove:Water 1:10	EO	83.39	1.34	0.21	14.34
Microwave-assisted SD inside [21]	10 min 500 W 100 °C Clove:Water 1:5	EO	67.54	18.33	0.02	10.59
Microwave-assisted SD outside [21]	10 min 500 W 100 °C Clove:Water 1:5	EO	56.06	34.15	-	4.69
SD [19]	240 min 100 °C Clove:Water 1:10	EO	82.65	0.91	0.13	15.63
SD [11]	600 min 100 °C Clove:Water 1:5	EO	48.82	36.94	4.41	3.89
SFE [14]	170 min SC–CO_2_ 40 °C 20 MPa	Organic extract	55.63	14.48	1.81	17.15
SFE [20]	14 min SC–CO_2_ 40 °C 15 MPa	Organic extract	55.44	7.77	0.86	12.53
SFE [11]	120 min SC–CO_2_ 30 °C 20 MPa	Organic extract	54.58	17.32	2.26	20.55
SFE [11]	120 min SC–CO_2_ 40 °C 30 MPa	Organic extract	55.14	15.52	2.02	20.32
SFE [11]	120 min SC–CO_2_ 50 °C 10 MPa	Organic extract	57.36	13.99	1.90	22.34
SFE assisted by cold pressing [20]	15 min SC–CO_2_ 40 °C 15 MPa 40 N.m	Organic extract	57.69	8.33	0.92	12.61
SFE assisted by cold pressing [20]	15 min SC–CO_2_ 40 °C 15 MPa 80 N.m	Organic extract	54.85	7.94	0.88	12.12
Soxhlet extraction [14]	720 min 69 °C Clove:Hexane 1:20	Organic extract	34.03	9.12	1.04	10.50
Soxhlet extraction [11]	360 min 100 °C Clove:Ethanol 1:8	Organic extract	57.24	1.75	2.03	19.37
Ultrasound-assisted SFE [14]	115 min SC–CO_2_ 40 °C 15 MPa	Organic extract	59.18	15.35	1.93	18.60

HD, hydrodistillation; SD, steam distillation; SFE, supercritical fluid extraction.

**Table 3:** Main food applications of CEO.

Food Category	Food	Application Form	Dose	Results	Reference
Baked foods	Cake, bread, green bean cake, and Buddha’s hand citron cake *	Storage	1%	Extended shelf life up to 2–12 days	[58]
	Bread *	Storage	250 mg/g	Extended shelf life up to 15 days	[66]
	Refrigerated steamed buns *	Coating	0–1.2%	Extended shelf life up to 10 days, but volatile components evaporate during the re-steaming process	[65]
Dairy products	Fresh soft cheese *	Fortification	0.01%	Extended shelf life up to 3 weeks	[65]
Meat, poultry, and seafood products	Fresh rainbow trout *^,+^	Coating		Extended shelf life up to 5–12 days	[67]
	Chicken breast meat *	Coating		Extended shelf life up to 12 days	[60]
	Beef sucuk *^,+^	Coating	1.50%	Extended shelf life up to 45 days	[68]
	Beef cutlets *^,+^	Coating	2 mg/g	Extended shelf life up to 12 days	[61]
	Sea bream *^,+^	Storage	10–15 mg/kg	Extended shelf life up to 15 days	[69]
	Bluefin tuna *^,+^	Coating	0.5 mL	Extended shelf life up to 14 days	[70]
	Ground beef *^,+^	Fortification	10%	Extended shelf life up to 7 days; at refrigeration and chilling temperatures 60 days Can produce unpleasant flavors	[63]
	Gelatin–chitosan film, Cod fillets *	Coating	15%	Extended shelf life up to 12 days; improved mechanical, structural, and barrier properties	[71]
	Raw grass carp fillets ^+^	Coating	0.1–1.0%	Reduced content of off-odor volatiles for 12 days	[72]
	White shrimp *^,+^	Coating	0.25–0.5%	Extended shelf life up to 15 days and inhibited melanosis	[73]
	Salmon burgers *^,+^	Fortification	0.005–0.01%	Extended shelf life up to 14 days and inhibited melanosis	[74]
	Chicken patties *^,+^	Coating	0.50%	Extended shelf life up to 35 days and inhibited melanosis	[75]
	Chicken breast *^,+^	Storage	0.2–0.5%	Extended shelf life up to 15 days and inhibited melanosis	[76]
Packaging material	Mechanically deboned chicken meat protein film *^,+^	Fortification	1%	Improved antioxidant and antimicrobial properties	[77]
	Poly (lactic acid) biocomposite food packaging film *	Fortification	3%	Improved antimicrobial properties	[78]
	Polylactide/poly(ε-caprolactone)/zinc oxide/CEO and scrambled eggs *	Fortification	25%	Extended shelf life up to 21 days, improved mechanical, structural, and barrier properties	[79]
	Chitosan–gum Arabic film *	Fortification	5%		[80]
	Citrus pectin film *^,+^	Fortification	0.5–1.5%	Improved barrier, mechanical, antioxidant, and antimicrobial properties of pectin film	[81]
	Chicken eggs *	Storage	10–80 µg/g	Extended shelf life up to 30 days, less weight reduction	[82]
Processed food	Ketchup *	Fortification	500 ppm		[64]
	Sausages *	Fortification	2000 mg/L	Prolonged shelf life for 14 days	[62]
Vegetables	Mango (cv. Banganapalli and cv. Totapuri) *^,+^	Storage	106 μL	Extended shelf life up to 20–21 days	[83]
	Persimmon *	Storage	1.56%	Inhibited mold growth on persimmon fruits for 28 days	[84]
	Pak choi *	Storage	0.02%	Extended shelf life up to 17 days	[85]
	Avocado *	Coating	0.20%	Extended shelf life up to 7 days	[86]

Bioactivity: * antimicrobial; ^+^ antioxidant.

**Table 4:** Principal biological activities of CEO.

Pharmaceutical Form	Bioactivity	Mechanism	Model	Dose	References
Clove essential oil ^C, HD, SD^	Analgesic	Mediation through opioidergic and cholinergic systems Inhibits voltage-gated Na^+^ channels and activation of TRPV_1_	Adult male Wistar rats [28] Yellowtail clownfish *Amphiprion clarkia* [88]	40–500 µL/L	[28,88]
	Anesthetic	Inhibits voltage-gated Na^+^ channels and activation of TRPV_1_ Reduces contraction of dorsal muscle	Wistar rats [28] Cardinal tetra *Paracheirodon axelrodi* Angelfish *Pterophyllum scalare* [89] *Cherax quadricarinatus* [90] Adult male Tilapia del Nilo *Oreochromis niloticus* [91]	50–500 μL/L	[28,89,90,91]
	Anticancer	Decreases levels of inflammatory biomarkers Inhibits tissue remodeling in protein molecules Inhibits pro-inflammatory genes and proteins such as pro-inflammatory cytokines Cytotoxic Genotoxic Induces apoptosis Antiproliferative activity Growth inhibition Changes polarization of cancer cells Inhibits proton pumps and ATP production	Human dermal fibroblasts [87], cancer cell lines (cervical, liver, breast, prostate, colon, erythroleukemia, lung) [92,93,94,95,96,97]	13–127 μg/mL	[87,92,93,94,95,96,97]
	Anticoagulant	Delays time for blood coagulation	Male Swiss mice (*Mus musculus*) [98]	0.0625–4 mg/mL	[98]
	Antidiarrheal	Ability to balance gut microbiota Helps intestinal motility Potentiates digestive process due to its ability to increase enzyme activity and nitrogen absorption Regulates neurotransmitters such as histamine and dopamine; Ca_2_^+^ activates Cl channel inhibitor TMEM16A, causing reduced intestinal motility in mice	Male Swiss mice (*M. musculus*) [98]	50–100 mg/kg	[98]
	Anti-inflammatory	Inhibits release or synthesis of inflammation-mediating compounds Decreases levels of inflammatory biomarkers Inhibits tissue remodeling proteins Inhibits level of expression of genes and proteins, pro-inflammatory proteins such as cytokines Inhibits prostaglandin synthesis and neutrophil chemotaxis Inhibits factor NF-kB in activation of tumor necrosis factor-*α* (TNF-*α*) Inhibits expression of cyclooxygenase (COX-2)	Rats [99] Human dermal fibroblasts [87] BALB/c mice [26]	100–250 mg/kg	[26,87,99]
	Antimicrobial	Inhibits growth Destabilizes membrane permeability and integrity Ruptures phospholipid membrane, resulting in electron transport inhibition, protein translocation, phosphorylation, and other enzymatic activity, leading to cell death	*Candida albicans*, *Klebsiella* spp., *E. coli*, *Proteus* spp., *Pseudomonas aeruginosa*, *Agrobacterium tumefaciens*, *Erwinia* spp., *S. aureus*, *Listeria innocua*, *Bacillus subtilis*, *Bacillus cereus*, *Listeria monocytogenes*, *Salmonella typhimurium*, *Lactobacillus acidophilus*, *Lactobacillus reuteri*, *Lactobacillus casei*, *Lactobacillus rhamnosus*, *Aspergillus niger*, *Tetrahymena pyriformis* [92,96,97,99,100]	1.25–6.25 mg/ mL	[92,96,97,99,100]
	Antinociceptive	Inhibits COX-2 and transient vanilloid receptor potential (TRPV) by high-voltage inhibition of Ca_2_^+^ currents in primary neurons	Female Wistar rats [101]	100 μg/kg	[101]
	Antioxidant	Radical scavenging activity Inhibits lipid peroxidation Transfers electrons or hydrogen atoms to neutralize free radicals and block oxidative processes Protective effect on ROS-induced biochemical changes and histopathological damage, balance between oxidant/antioxidant ratio	DPPH, *β*-carotene-linoleate, ABTS, FRAP, Folin–Ciocalteu, flavones and flavonols, flavonoids, TAC [93,97,99,100,102]. Wistar rats/blood, histopathological study [102]	30–600 μg/mL	[93,97,99,100,102]
	Antipyretic	Reduces chemotaxis Inhibits COX-1 and COX-2	Male Swiss mice (*M. musculus*) [98]	50–100 mg/kg	[98]
	Hemolytic	Interacts with the cell membrane	Male Swiss mice (*M. musculus*) [98]	0.625–2.5 mg/m	[98]
	Insecticide Contact toxicity Repellent Larval toxicity Oviposition deterrence	Inhibits life cycle Inhibits development Attacks three possible molecular targets (transient receptor potential (TRP) channels, octopamine receptors, and gamma-aminobutyric acid (GABA) receptors) Neurotoxic action Increases cell membrane permeability, breaking cytoplasmic membrane and interacting with proteins Hydroxyl group present in eugenol binds to proteins and affects their properties Inhibits enzymes ATPase, histidine decarboxylase, amylase, and protease Absorption by cuticular lipids, then enters hemocoel and nervous system, or tracheal system absorbs it	*Ctenocephalides felis felis, Rhopalosiphum maidis, Coccinellidae, Coleomegilla maculate, Culex pipiens, Blattella germanica, Ae. j. japonicus* [103,104,105,106,107]	4 mL/cm 5–80 mg/L	[103,104,105,106,107]
Microemulsion ^SD nmslyyds^ 303 nm Montanov 202™ Phase inversion method	Anti-inflammatory	Re-epithelialization and formation of dermis and epidermis. increases collagen synthesis	m5S cell line Male Wistar rats [27]	0.2 g	[27]
Nanoemulsion ^C nmslyyds^ 6–27 nm Tween 20 and 80 Spontaneous self-emulsification	Antimicrobial	Destabilizes membrane permeability	*S. aureus* [108]	19–24 μg/m	[108]
	Anticancer	Antiproliferative effect Cytotoxic activity Induces necrosis	Thyroid cancer cell line [108]	19–24 μg/mL	[108]
Nanoemulsion ^C nmslyyds^ 29.1 nm Tween-80 Spontaneous self-emulsification	Wound healing	Reduces wound epithelialization period Increases leucine content Increases collagen content Induces neovascularization	Female albino Wistar rats [109]	0.61 mg/g	[109]

Source: ^C^ commercial CEO, ^SD^ steam distillation, ^HD^ hydrodistillation.
